# Design and Implementation of a Personalized Tourism Recommendation System Based on the Data Mining and Collaborative Filtering Algorithm

**DOI:** 10.1155/2022/1424097

**Published:** 2022-08-31

**Authors:** Xiang Nan, Kayo kanato, Xiaolan Wang

**Affiliations:** ^1^Nantong Normal College, Nantong 226000, Jiangsu, China; ^2^Department of Tourism It Research, Kyoto University of Information Technology, Kyoto 602-0057, Osaka, Japan; ^3^College of Foreign Languages, Guangdong University of Science and Technology, Dongguan 523083, Guangdong, China

## Abstract

A personalized tourism recommendation system provides convenient and economically affordable travel information for individuals/groups. This recommendation system banks on accumulated and analyzed data for providing context-aware travel solutions. For improving the recommendation efficiency and data analysis of such systems, this article introduces a mining and filtering harmonized collaborative process, named as the collaborative mining and filtering process (CMFP), for reducing the data processing overheads and improving the recommendation ratio. In this process, the accumulated data from the global and personal travel, expenditure, and other information are collaboratively analyzed. This analysis is powered by knowledge-based transfer learning for reducing the retardation in the large data processing. Based on the context-based data analysis, the filtering and mining are jointly performed for providing recommendations. In the filtering process, the maximum processed contextual data are extracted for updating the current knowledge base. From this base, the recommendation for adaptable travel is recommended for the user. This process's performance is analyzed using the metrics accuracy, data handling rate, mining time, and overhead.

## 1. Introduction

A personalized tourism recommendation system is a process that recommends important places and spots to tourists. The tourism recommendation system provides various services such as the preferences and interests of users. A personalized tourism recommendation system analyses the visited places of users and recommends unvisited places [1]. Understanding customers' preferences and interests plays a major role in the tourism recommendation system. User's profile-based personalized tourism recommendation system is widely used among customers [2]. The main focus of personalized tourism recommendation systems is to evaluate users' physical and mental states by analyzing their condition of users. Various filtering techniques and methods are used in a personalized tourism recommendation system that provides necessary information about users [3]. Collaborative filtering is the most commonly used technique that identifies the key factors and values of a user's interest. The collaborative filtering technique provides proper recommendations for customers that improve the performance and efficiency of tourism recommendation systems. Users' behaviors, interesting places, and uninterested things are identified by a personalized tourism recommendation system [4, 5].

Data mining is a process that identifies necessary information from large amounts of data and provides the necessary set of data for the further analysis process. Tourism data mining is used to analyze the number of tourists that are arrived at a particular place [6]. Tourism data mining also analyses the profiles of customers and expenditures of tourists for the recommendation system. Data mining provides necessary services for a tourism management system that improves the performance and effectiveness of the system [7]. Tourism data are collected from various analyses and management processes. Tourism data mining provides various strategies and techniques to ensure the safety and security of users while traveling. The data mining process improves the communication services for the tourists that reduce the latency rate in the searching process [8]. Tourism filtering is a process that delivers travel information to tourists. Information such as travel advice, guide, travel planning, and expenditure is produced by a filtering technique. The collaborative filtering (CF) technique is mostly used in tourism recommendation systems [9]. CF provides appropriate options and choices for tourists that improve the recommendation system. The CF method searches for a smaller set of users with similar preference among larger people and guides the tourist to the particular areas. CF reduces unwanted problems and unnecessary worries while traveling [10]. A significant amount of tourism information is offered to users, but at the same time, it has led to the blind decision of users. Massive data have overloaded the information that people are genuinely interested. The introduction of tourist recommendation systems helps consumers tackle this challenge. Against the foregoing backdrop, this study proposes and executes a tourist recommendation system based on data mining. From the standpoint of mining the similarity between users, the similarity between users is computed by the collaborative filtering algorithm, and then, the attractions visited by people with higher similarity are suggested.

Machine-learning (ML) techniques are most widely used in various fields to improve the accuracy rate in detection, analysis, and prediction processes. The ML technique also improves the performance and efficiency of the system [11]. The tourism data analysis process mostly uses ML techniques to enhance the effectiveness of the system. The tourism data analysis is a process that identifies the important and key areas or fields that are needed to improve the tourism recommendation system [12]. The tourism data analysis also analyses the user's behaviors, interests, likes, and preferences to provide necessary services at the needed time. The support vector regression (SVR) model is an ML-based model that is used in the tourism data analysis process [13]. SVR improves the accuracy rate in the analysis process, which enhances the feasibility of the recommendation system. SVR collects necessary data from the analysis process and produces an optimal set of data for the recommendation system. SVR analyses various information such as photographs, texts, videos, forecasting details, and maps, and finally provides appropriate details for the tourists. SVR predicts the accurate information and details that are needed for the analysis process. The multilayer perceptron (MLP) model is also used in the tourism data analysis process. MLP reduces the error rate in the analysis process, which improves the significance and efficiency of the tourism recommendation system [14, 15].

As a result of this analysis, SVR provides travelers with the information based on the informed decisions about their travel plans. Further, the numerical validation has been estimated based on the SVR, which helps to predict the information required. For tourist data analysis, the higher accuracy rate in the analytical process validates the recommendation system, which is likely to succeed. Besides to provide a recommendation system with the best possible data, SVR gathers the essential information from its analysis.

## 2. Related Works

Li et al. [16] introduced a forecasting model for tourism demand using social network data. A gradient boosting regression tree is used here to find out the weather, holidays, condition, and location of an area. The proposed model provides a better data analysis process that increases the effectiveness of the system. The proposed model is mainly used for improving the accuracy rate of the tourism demand forecasting system. When compared to other models, the proposed forecasting model increases the feasibility of the system.

Ahmad et al. [17] proposed a stochastic approach using the Markov chain model for travel route optimization. The Markov chain model is used here for the prediction process that identifies both long- and short-term bases. The proposed method finds out the location and condition of a place and produces the necessary set of data for further process. User constraint data are used here for the prediction and detection process. The proposed approach increases the accuracy rate in the detection process, which enhances the performance of the system.

Migliorini et al. [18] introduced an adaptive trip recommendation system for tourism. The proposed approach increases the point of interest (POI) in a recommendation system. The main aim of the proposed approach is to focus on improving the balance among POI. The proposed approach reduces the overall optimization problems that improve the effectiveness and efficiency of the system. Experimental results show that the proposed approach increases the accuracy rate in the tourism recommendation system.

Yang et al. [19] proposed a hierarchical multi-clue fusion (HMCF) model POI prediction process in the tourism system. The user-generated content (UGC) is mostly used in HMCF that identifies the important features for the prediction process. HMCF predicts the actual POI and produces necessary information for the travel recommendation system. The proposed HMCF model increases the accuracy rate in the POI prediction process, which improves the significance and performance of the system.

Wang and Tang [20] introduced a sentiment-aware multimodal tropic model (SMTM) for a personalized travel recommendation system. The proposed SMTM analyses the tropic information and provides an appropriate set of data for the recommendation system. SMTM finds out the sentiments that are given by the tourists and produces an actual set of data for the analysis process. When compared to other models, the proposed SMTM improves the effectiveness and feasibility of a personalized travel recommendation system.

Alrasheed et al. [21] proposed a multilevel tourism recommendation system that finds out the destination for the customers. The proposed system identifies travelers with similar interests and recommends the destinations. The recommendation system also provides various similar destinations for the customers, which enhances the performance of the system. Key interests, preferences, and values are identified using a data analysis process that plays a major role in improving the services. The proposed system improves the quality of services in the travel recommendation system.

Abbasi-Moud et al. [22] introduced the context-aware fuzzy ontology method (CAFOB) using a tourism recommendation system. The proposed method analyses the reviews that are given by tourists. The CAFOB identifies the weather condition, time, location, and direction of destination that increase the quality of services in the travel recommendation system. The ontology-based method improves the accuracy rate in the detection process, which enhances the feasibility and effectiveness of the system.

Hong and Jung [23] proposed a new model for a tourism recommendation system, named the multicriteria tensor model. Points of interest, preference, reviews, and ratings are first analyzed by the proposed model. The collaborative filtering (CF) technique is used here to find out the key factors that are needed for the recommendation system. Experimental results show that the proposed model improves the stability and performance of the tourism recommendation system.

Zhang and Tang [24] introduced a tourism spot recommendation system using a neural network (NN) approach. The NN trains the dataset and provides an optimal set of data for the prediction process in the travel recommendation system. Potential features are identified by the deep NN approach that reduces the latency rate in the computation process. The proposed method improves the accuracy rate in the prediction process, which increases the effectiveness and feasibility of the system.

Zheng et al. [25] proposed a new tourism recommendation system based on users' sentiments. User preference, interests, and destination popularity are identified using temporal dynamics. The proposed model finds out the popularity by analyzing the reviews that are produced by travelers. User sentiment plays a major role in the recommendation system that reduces the time consumption rate in the searching process. When compared to other models, the proposed recommendation system increases the accuracy rate in the recommendation system, which improves the performance and quality of the system.

Logesh et al. [26] introduced a sentiment analysis-based tourism recommendation system. Both convolutional neural networks (CNN) and the long short-term memory (LSTM) approach are used here to improve the quality of services in the recommendation system. The LSTM identifies the popularity among the destination based on reviews that are given by the tourists. The CNN analyzes the necessary set of data and produces appropriate information for the tourism recommendation system. The proposed model increases the efficiency and effectiveness of the recommendation system.

Shi [27] proposed a tourism demand forecasting method based on a neural network (NN) algorithm. The NN extracts the important features and factors that are needed for the forecasting process. Preference and influencing factors are identified by understanding certain demands in the tourism system. Both the accuracy rate and an efficiency rate of the recommendation system are improved using the data mining process. The proposed method increases the performance of the rural-based tourism recommendation system.

Cheng [28] introduced a new travel route commendation system using an interesting theme and a distance matching technique. The proposed model first finds out the user's interests and preferences and then suggests a destination. The travel route calculation method is used here to calculate the destination and provide necessary information for the travelers. Both time and direction of the destination are calculated by performing a prediction process. The proposed model increases the accuracy rate in the prediction process, which improves the efficiency and feasibility of the recommendation system.

## 3. Proposed Collaborative Mining and Filtering Process

The proposed mining and filtering process in the personalized tourism recommendation system is designed and implemented to improve the recommendation efficiency and data analysis of the travel information for individuals or groups. It regulates through the recommendation system that banks on accumulated and analyzed data for better accuracy providing context-aware travel solutions.

To learn about a user's tastes, the algorithm examines his or her past browsing history. It divides the users into groups based on their distinct preferences, and then suggests things that other members of the group will enjoy. On the basis of the above information, this article creates a trip recommendation app using data mining, including popular attraction ranking based on collaborative mining and filtering process search results.

**Figure 1:**
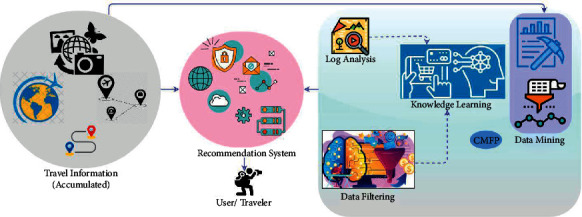
Proposed recommendation system.

The proposed CMFP model gathering data from the personal travel, global, and expenditure information is provided to control the data processing overhead in the tourism recommendation system. In the tourism recommendation system, some additional context data such as social media sentiment, weather, user preference, time, and location information are analyzed for providing more accurate tourism recommendations.  Figure 1 presents the proposed recommendation system.

The tourism recommendation is a combination of data mining and a collaborative filtering process to analyze the travel database accumulated from the tourism recommendation system. The travel history is provided with travel database recommendations to store and retrieve the information that relies on user preference and contextual conditions. In the proposed method, reliable tourism recommendation and data analysis of such systems are devised by reducing the retardation based on the large data processing. The context-based data analysis depends on the own preference and varying contextual conditions that can be analyzed and then recommended to the users. The knowledge of places and their locations is prominent for the user who will need data and directions for each recommended tourist place around their current location. The process of the tourism recommendation system is regulated using online websites, mobile applications, and nearby tourism recommendation offices. The CMPF method is based on filtering and data mining process from the accumulated data; CMPF process ensures the tourism information is collaboratively analyzed through regression knowledge-based transfer learning for reducing the retardation in the vast amount of data processing. A collaborative mining and filtering process is used to predict tourist demand, which is compared with a strategy that uses data information, such as the destination price level or the Web search traffic per sending country, to estimate arrivals. This research relies on collaborative mining and filtering process to make predictions effectively.

The tourism recommendation provides travel history from the accumulated data inputs based on large travel information (*Tr*_inf_) observed in the travel database. The tourism recommendation is provided to make decisions based on what the traveler/user has previously rated and what the user is currently looking at, using knowledge-based transfer learning. The convenient and economically affordable travel information for individuals/groups is aided for providing a reliable recommendation from the gathered large number of data. This decision-making process based on relevant contextual data should be considered for a large amount of data processing; the issues can be addressed by mitigating the collaborative filtering process and data mining process that outputs in data processing overheads where the tourism information is initially filtered, and by mining the harmonized collaborative process. The input travel information is computed as follows:

(1) $$\begin{array}{c} Tr_{\inf}=\frac{1}{L}\left|\sum_{c=1}^{t}D_{M}\left(c\right)-C_{F}\left(c\right)\right|. \end{array}$$

In formula (1), the variables *D*_*M*_(*c*) and *C*_*F*_(*c*) represent the travel information based on data mining and collaborative filtering for the time interval *t*, and the variables *c* and *L* are the contextual information and a large number of data, respectively. If *c* and *L* information for individuals/groups is based on the user preference and then *c* ∈ [0, *∞*] and *L* ∈ [−*∞*, 0], hence, the following is obtained:

(2) $$\begin{array}{c} D_{M}\left(t\right)=\frac{1}{c}\int_{-\infty }^{\infty }\frac{D_{M}\left(c\right)}{t}\text{d}t,\\ C_{F}\left(t\right)=\frac{1}{c}\int_{-\infty }^{\infty }\frac{C_{F}\left(c\right)}{t}\text{d}t. \end{array}$$

In (2), the travel information is suppressed for all *D*_*M*_(*t*)+*C*_*F*_(*t*), which denotes a large number of data based on travel history rely on *c* and *L* for the different time intervals on (*f* × *t*). Here, *f* is the fetching process. The fetching process is performed to reduce the handling rate process in tourism management. The travel information is recommended to the individuals/groups based on the travel database information accumulated in the tourism recommendation system at any interval  *t*. Many ideas and approaches are provided by tourism data mining to keep people safe and secure when they are traveling. The communication services for visitors are improved by the data mining method, which reduces the latency rate in the search process. The technique of delivering travel information to visitors is called tourism filtering. Filtering is used to generate several types of travel-related data, including tips, guides, itineraries, and costs.

This personalized tourism recommendation system follows a high data handling for fetching of travel information from a large number of data to a small data, which is estimated as follows:

(3) $$\begin{array}{c} \left.\begin{array}{c} D_{M}\left(t\right)=\frac{D_{M}\left(c\right)}{t}*2^{\sqrt{2}}.c_{i}\left[R\left(e\right)*f+t-L^{2}\right]\\ \text{such that}\\ C_{F}\left(t\right)=\frac{C_{F}\left(c\right)}{t}*2^{\sqrt{2}}.c_{j}\left[D\left(a\right)*f+t-L^{2}\right] \end{array}\right\}. \end{array}$$

**Figure 2:**
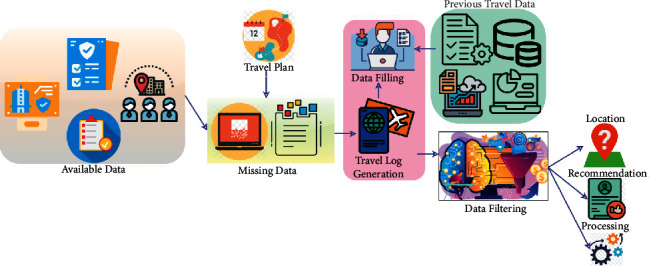
Data fetching and filtering.

In (3), the variables  *c*_*i*_ and  *c*_*j*_ are the fetching of travel information from a large number of data to a small data for better tourist place recommendations to the user based on their preferences. Reduced handling rates are achieved in tourist management through the use of a “fetching” technique. In the tourist recommendation system, the trip information is suggested to individuals and groups at any interval *t*, depending on the travel database information. As a result, this system uses a large amount of data to get trip information that is judged to be accurate as shown in (2) and (3). The factors of *R*(*e*) and *D*(*a*) represent the recommendation efficiency and data analysis based on fetching travel information. The data fetching and filtering process are presented in Figure 2.

Based on the available data, the travel plan is analyzed for its missing sequences. The missing data are filled using the generated travel log, i.e., [*T*_*r*inf_] ∈ *D*_*M*_ and the previous travel data. The filtering is based on *C*_*F*_(*t*) such that different functions such as location, recommendation, and processing are performed (refer to Figure 2). From the instance of providing partial data mining or contextual information filtering, the efficiency of input recommendation is computed and data analysis is performed, but the additional contexts have been ignored. The large travel information performs both collaborative filtering and data mining. Here, the recommendation efficiency based on *Tr*_inf_ is defined as follows:

(4) $$\begin{array}{c} Tr_{\inf}\left[R\left(e\right)\right]=\frac{2^{\sqrt{2}}\left[\left(f\times sL\right)-D\left(a\right)\right]}{t^{2}}\left[\;c_{i}-\;c_{j}\right]. \end{array}$$

In (4), the data processing overhead in the travel history database condition *Tr*_inf_[*R*(*e*)] is analyzed after fetching the information. From this tourism management based on less *Tr*_inf_[*R*(*e*)], the accumulated data from the global, expenditure, and personal travel information are extracted for further recommendation, where *ov* is the data processing overhead occurrence in the tourism recommendation processing, and the variables *e*_*h*_ and *e*_*L*_ are the high and low recommendation efficiency in *Tr*_inf_. The log travel recommendation of *Tr*_inf_ and *R*(*e*) based on the *f*(*t*) is computed as follows:

(5) $$\begin{array}{c} R\left(e\right)\left[Tr_{\inf}\right]=\frac{f\left(t\right)}{\log\left[t/e_{h}-e_{L}\right]}. \end{array}$$

In (5), decision-making can be aided by recommendation systems, which provide users with a list of items based on their preferences. Automated systems that alter their recommendations based on their surroundings are known as “context-aware recommendation systems.” In order to make suggestions that are relevant to the user's current situation, the system uses a method called context matching to compare ratings from different situations.

This tourism recommendation system is computed for collaborative process *R*(*e*)[*Tr*_inf_], and *f*(*t*) is alone with the data processing at different time intervals. The context-based data analysis in the travel recommendation system is based on *D*_*M*_(*Tr*_inf_) and *C*_*F*_(*Tr*_inf_) using knowledge-based transfer learning. From each travel database, the collaborative filtering process is retrieved, followed by the joint performance of all participants. Here, the contextual information is processed based on the trip suggestion; an extensive data processing using prior knowledge of location and context data is used as shown in (6) and (7).

This large data processing based on user recommendation helps to maximize the data mining by improving the recommendation ratio of varying recommendation system banks for all *D*_*M*_(*t*) and *C*_*F*_(*t*) providing context-aware travel solutions. In this context-aware travel data analysis, the collaborative filtering process is extracted from each travel database followed by the joint performance. The role of contextual information will be quantified, and the aid of recommendation techniques will be identified. The first pillar of travel recommendation is determined as a large data processing based on the previous knowledge about the location and context data in the following equations:

(6) $$\begin{array}{c} R\left[Tr_{\inf},\;t\right]=-\sum_{i=1}^{f}c-\sum_{j=1}^{t}t-\sum_{i=1}^{f}\sum_{j=1}^{t}D_{Mi}C_{Fj}, \end{array}$$

where

(7) $$\begin{array}{c} L\left[Tr_{\inf},\;t\right]=\frac{ov^{-R\left[Tr_{\inf},t\right]}}{{\sum_{i=1}^{f\times t}ov}^{-R{\left[Tr_{\inf},t\right]}_{ij}}}. \end{array}$$

**Figure 3:**
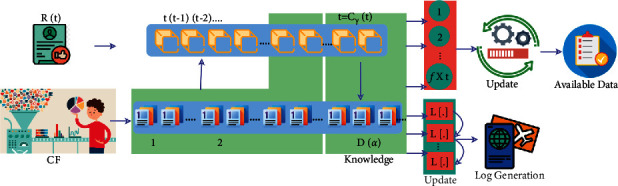
Data analysis for filtering.

Based on (6) and (7), the collaborative filtering and data mining processes of the knowledge base are used such that *R*(*e*)[*Tr*_inf_,  *t*] is evaluated for providing a better recommendation for the users based on the condition ∈*R*(*e*)[*Tr*_inf_]. This context-based data analysis helps to determine whether the tourism recommendation system based on fetching the information to ease possible travel information is collaboratively analyzed. The collaborative data analysis for filtering output is presented in Figure 3.

The *R*(*t*) and *C*_*F*_ are the inputs for the knowledge-based learning as presented in Figure 3. The states are divided as [*t* − *C*_*F*_(*t*)] ∈ *D*(*α*) (end) provided *c* ∀  (1, *f* × *t*) is mapped. If  *t* to (*t* − *x*)∀  *x* < [*t* − *C*_*f*_(*t*)] is observed in *D*(*α*), then *L*[.] is continuously updated. This represents the alternating state for which log generation ∈*C*_*F*_ is performed. Based on the (*f* × *t*) interval, the available data are filtered for maximizing accuracy. The recommendation efficiency was calculated using transfer learning based on *R*(*e*)[*Tr*_inf_ ∈ [−*∞*,  *∞*]] and *D*_*M*_ ∈ [0, *∞*] (or) *D*_*M*_ ∉ [0, *∞*]. The condition for mining data succeeds a large data processing of [−*∞*,  0] that time retardation is addressed in data analysis at regular time instance  *t*. The data processing of *R*(*e*)[*Tr*_inf_] is individually or jointly performed using *L*[.] wherein a large number of data processing instances *L*[.]^*∗*^ illustrate the condition of *D*_*M*_ ∈ [0, *∞*] is recommended, and therefore, the travel history *L*[.] does not handle further travel recommendations. The above contextual information variation analysis, *D*_*M*_ ∈ [0, *∞*], is performed as the data processing *C*_*F*_ ∉ [0, *∞*], and therefore, the context-aware data are considered. This data processing overhead (*ov*) is mismatched with the data mining and collaborative filtering processes to require the context-aware travel solution (*Tr*_*S*_). To ensure that travel information is cost-effective for users, collaborative data validation is the determining element. As a result, the performance of retrieving vast amounts of information at a high data processing rate at various time intervals based on recommendation efficiency is processed. Based on mining data, a collaborative filtering technique is used to identify the initial overhead. The collaboration process is the main factor that helps to analyze the trip information based on data processing overhead validation. Therefore, the efficiency of a tourist recommendation system is measured by the amount of information that can be retrieved in a given amount of time.

Hence, the data mining and filtering process outputs in {*c*_1_toc_*f*×*t*_} are computed. The collaborative filtering and data mining instance outputs along with high accuracy *A*_*c*_ are estimated as follows:

(8) $$\begin{array}{c} \left.\begin{array}{c} c_{1}=R\left(e\right)\left[Tr_{\inf}\left(1\right)\right]\\ \vdots \\ c_{f\times t}=R\left(e\right)\left[Tr_{\inf}\left(f\times t\right)\right]-\frac{A_{cf\times \left(t-1\right)}}{R{\left[Tr_{\inf},\;t\right]}_{f\times \left(t-1\right)}} \end{array}\right\}. \end{array}$$

In (8), the travel information for individuals/groups at which *R*(*e*)[*Tr*_inf_(*t*)] is determined from *R*(*e*)=*D*_*M*_(*c*)+*C*_*F*_(*c*) to *D*_*M*_(*c*) or *C*_*F*_(*c*) is alone processed. If the affordable travel information provided to the users is based on data processing overhead validation, the collaborative process is the equating factor. Therefore, tourism recommendation efficiency is the performance of fetching large information to data handling rate at a different time interval. The first overhead is identified based on mining data, and a collaborative filtering process is performed. The accuracy is computed as follows:

(9) $$\begin{array}{c} A_{c}=\frac{1}{L}\left[\frac{c_{f\times t}\in D_{M}}{c_{f\times t}\in \left(D_{M}+C_{F}\right)}+\frac{c_{f\times t}\notin D_{M}}{c_{f\times t}\in \left(D_{M}+C_{F}\right)}\right]. \end{array}$$

In (9), the condition based on data mining and collaborative filtering process represents the accumulated data such that *c*_*f*×*t*_ ∉ *D*_*M*_ or *c*_*f*×*t*_ ∈ *D*_*M*_ ≤ 0 < *c*_*f*×*t*_ ∈ (*D*_*M*_+*C*_*F*_) is the reliable context-aware travel solution for mining and filtering data *R*(*e*)[*Tr*_inf_]. If the above-derived condition is not satisfied, then the data processing overhead increases. Similarly, if *c*_*f*×*t*_ ∉ *D*_*M*_ is required for updating the current knowledge base, then *ov*=*ov*+1 else data handling rate such that *dh*=*dh*+1. In travel information analysis, it is important to increase the data handling rate other than the previous data processing overhead. For this assimilation, both data mining and collaborative filtering are performed for all the context-based data analysis output in the above condition. The overhead identification in a large number of data processing with the accumulated and analyzed data assists to calculate the occurrence of  *ov*, *dh*, and mining time; this series estimation helps to improve the accuracy of travel information.

Collaborative filtering (CF) is employed here to identify the most important factors in a recommendation system's algorithm. The suggested model increases the stability and performance of the tourism recommendation system, according to experimental data. Using a recommendation system built on a base of collaborative filtering and data mining, trip planning is more precise than ever before.

Personal preferences and fluctuating contextual variables may be used to interpret the data, which can then be recommended to users. The user will need information and instructions for each of the recommended tourist destinations in the vicinity of their present location. In order to manage the tourism recommendation system, Internet websites, mobile applications, and nearby tourism recommendation offices are used to control it. In this context-based data analysis and further mining and filtering process computing, the goal is to provide better recommendations for the user to travel to the best place and also provide information about tourist places around their location, and contextual information is to reduce the data processing overhead, and therefore, the travel database is stored, and retrieved travel history for other users providing recommendations *R* is given as follows:

(10) $$\begin{array}{c} R\left(f\right)=R_{e}\left[\frac{\left(C_{F}\times ov/dh\right)}{Tr_{\inf}\left(c\right)-D_{M}\left(t\right)*ov/dh}\right]. \end{array}$$

In (10), the recommendation system utilizes a user-based collaborative filtering mechanism to validate the process. It indicates that suggestions are offered to the target user based on the similarity between the profiles of the users. Each user's preferences, travel history, and search history are all recorded in a database, and suggestions are generated based on the information in the database. Prior to making a recommendation, the travel database must locate a nearby user who shares the target user's preferences. The neighbourhood estimator makes use of the user profile and rating data from the user database to do this.

**Figure 4:**
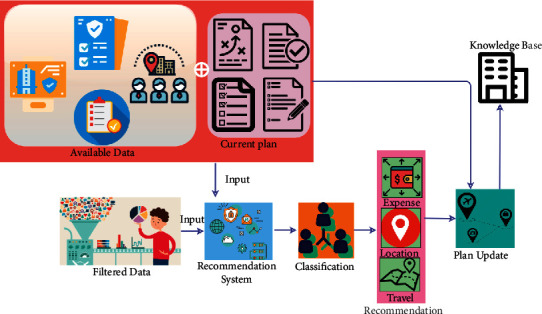
Recommendation system after the data analysis.

The equation (10) represents the tourism recommendation system for the individuals or groups based on the data processing instances. The travel history provided based on the user preference and contextual information using the knowledge-based transfer learning process is either collaborative filtering or data mining, in both conditions, if *Tr*_inf_(*c*)=0, then the fetched large number of travel information is suppressed to a small number and is performed as *Tr*_inf_=*C*_*F*_=*D*_*M*_, which is the recommendation maximizing condition based on the condition *Tr*_inf_(*c*)=1, otherwise *C*_*F*_=*D*_*M*_ − *Tr*_inf_. Therefore, the occurrence of data handling is a reliable output, whereas the travel history data based on locations and contextual information from the previous knowledge base (without overhead) are as mentioned in (1). This is accumulated and analyzed for all *Tr*_inf_ ∈ *t* and *D*_*M*_ ∈ *f* in (1). The tourism recommendation system in this scenario is processed for all transfer learning output, where the recommendation for adaptable travel is recommended for the user, and therefore, the recommendation instance is stable as in (1).  Figure 4 presents the recommendation system design using the analyzed data.

The recommendation system generates expense, location, and travel-based information. This information updates the actual plan and knowledge. The actual available data are filtered using the classification performed using transfer learning. The final recommendation is used for improving the knowledge-based input that is used for further analysis (refer to Figure 4). The computation of collaborative filtering and data mining based on tourism recommendations for all the time intervals, the user preference, and contextual information is changed based on that condition providing the recommendation of the user for fetching and extracting data. The real-time personalized tourism management system is available at different *t* instances during the context-aware travel solutions. From this knowledge base, the travel recommendation for adaptable travel is recommended for the traveler/user. The proposed CMPF balances collaborative filtering and data mining processes until a recommendation is computed, whereas different adaptable travel is recommended for the user preference and the maximum contextual information is extracted for updating the current knowledge base depending on user recommendation at any interval of time. Based on this condition, if the travel information and recommendation are provided for the individuals or groups based on their preferences, the minimum contextual data observation and extraction instance are observed. Therefore, the tourism recommendation system consecutively maximizes the travel history and database through transfer learning; it identifies the data processing overhead in the tourism management and recommends the best place to travel for the user through the knowledge-based transfer learning process. The travel history observation provides a different place and contextual information about the places. This tourism recommendation system under a context-based data solution is used to reduce the data processing overhead.

## 4. Discussion

**Figure 5:**
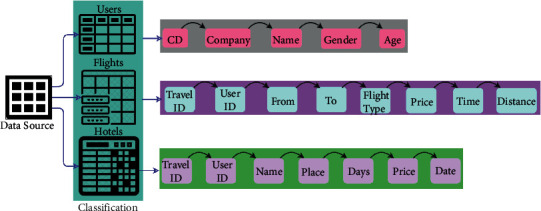
Dataset representations for analysis.

This discussion section analyses the proposed process's performance using experimental validations. The data source [29] was used for validating CMFP's performance. This data source provides (flights, hotels, and users) information for different travel destinations. This is observed as the available data; the analysis is presented for the representation as in Figure 5.

The representation in Figure 5 presents the utilizing data interconnected in each classification. The common information is used for tracking the travel history and providing information and recommendations. A new recommendation is augmented in the data source as an update. The primary goal of this research is to validate the information about a person's physical and psychological functionality while creating a user profile that affects the recommendation outcomes significantly. Here, the ability to accommodate visitors with various levels of physical and psychological impairments is validated based on the data interconnection as shown in Figure 5.

**Figure 6:**
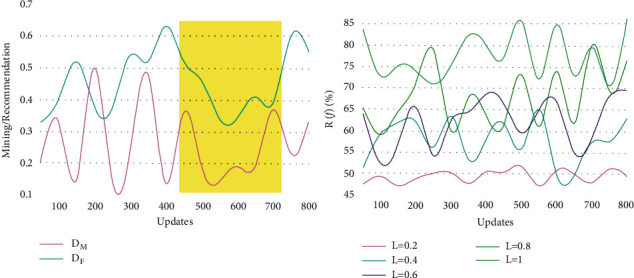
Mining/recommendation and **R**(**f**) % for different updates.

Therefore, for the varying updates, the mining/recommendation rate and *R*(*f*)% are analyzed using Figure 6.

**Figure 7:**
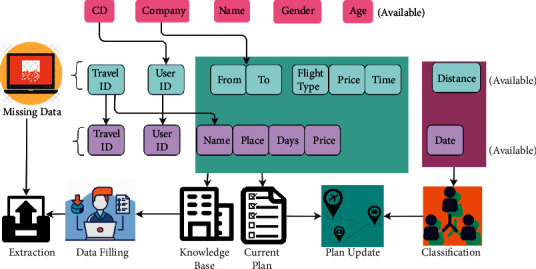
Filtering data representation.

The proposed process achieves a high mining rate/recommendation for *D*_*F*_ compared with that for *D*_*M*_. Based on *R*(*e*)[*Tr*_inf_] and *L*[*Tr*_inf_,  *t*], the *c* ∈ (1, *f* × *t*); the mining is performed consecutively ∀ *D*_*F*_. For any interrupts in *D*_*F*_, the *D*_*M*_ validation is performed for *R*[*Tr*_inf_, *t*] provided recommendations are high. This is stabilized past the classification process, and hence, filtering is required. In particular, for the augmented data (Update), the filtering is performed. Based on the learning knowledge, further recommendations are provided. As the data validations are high, the (*D*_*M*_+*D*_*F*_) generates maximum recommendations. The recommendation is performed (expected) after the classification as performed by the knowledge learning.  Figure 7 shows the representation of the filtering data from the input data.

In travel data analysis, speeding up data handling is more significant than reducing the prior data processing overheads for effective filtering. Data mining and collaborative filtering are used for every context-based data analysis output in this assimilation.

The cumulative data augmentation based on availability and classification is grouped as in Figure 7. In the representation, the classification based on distance and data is performed. The common data attributes other than flights and hotels are extracted as missing data. The knowledge base stores the travel user-related information with the precise locations based on distance, data, and days. Specifically, for the supplemented data (Update), the filtering is conducted. Based on the learned information, more recommendations are presented. As the data validations are high, it generates maximum recommendations. The suggestion is performed based on the categorization, which is performed by the knowledge learning.

Extraction of social connections based on the classification model helps to divide the dataset's users into male and female groups for assessment purposes because there is no explicit classification of users by gender in the dataset. Using the dataset's possible user data, it is divided into male and female user groups based on the classification ratio.

**Figure 8:**
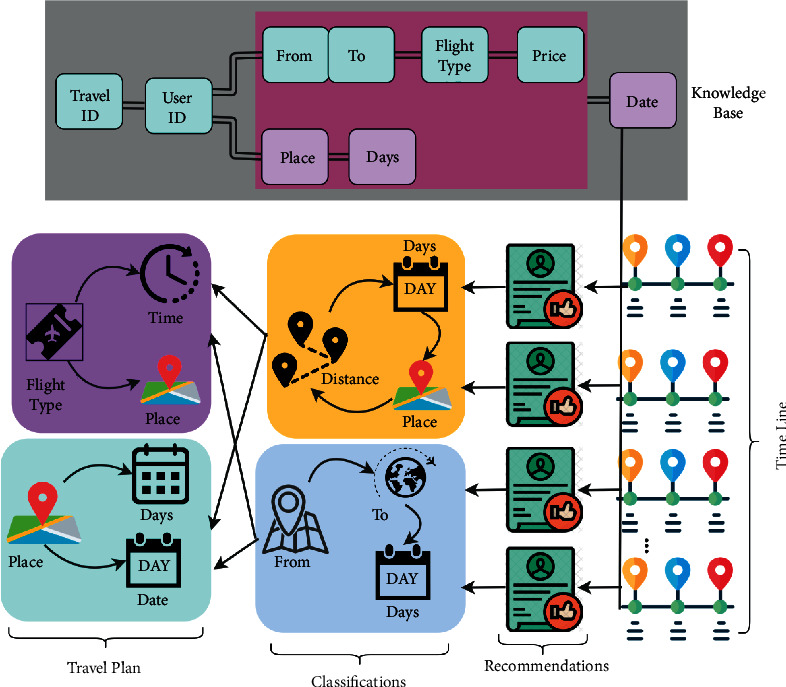
Recommendation-based representations.

If new data are augmented, the then current plan is changed and the knowledge base is updated. The recommendation-based representation for the above plan is illustrated in Figure 8.

The recommendation is based on different timelines as provided by the knowledge base. Here, *Tr*_inf_  is split into different classifications and travel plans for which *L*[ ] is performed. Depending on the information, filtering is performed, and hence, limited data are utilized. In this representation, the recommendations are valid until the new timeline is observed (refer to Figure 8). In the following subsection, the comparative analysis is presented. The comparative analysis for the metrics accuracy, data handling rate, mining time, and overhead is presented. In this analysis, the existing SMTM [20], ATRS [18], and CAFOB [22] methods are used.

## 5. Accuracy

**Figure 9:**
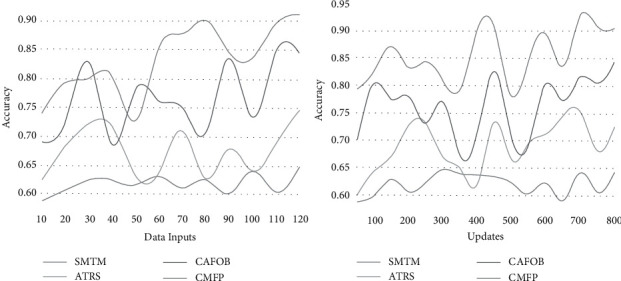
Accuracy analysis.

In Figure 9, the data processing overhead is detected from the tourism recommendation system that banks on accumulated and analyzed data for providing contextual information to improve the recommendation accuracy and provides convenient and economically affordable travel information using knowledge-based transfer learning. The filtering and mining are jointly performed for providing recommendations at different intervals. The travel information input is stored and retrieved from the travel database and travel history based on the contextual information and recommendation providing context-aware travel solutions wherein large information can be fetched into a small number. This overhead is addressed by collaborative process and data analysis of such systems based on the condition 1/*L*|∑_*c*=1_^*t*^*D*_*M*_(*c*) − *C*_*F*_(*c*)| satisfying successive tourism recommendations based on accumulated data from the global, preventing overhead. Therefore, the overhead identified in context-aware data analysis is reduced, preventing high accuracy due to adaptable travel recommended for the user.

### 5.1. Data Rate

**Figure 10:**
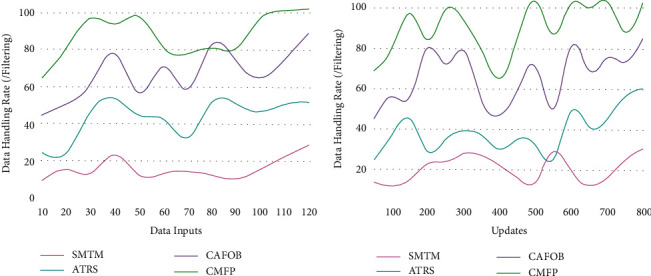
Data handling rate analysis.

The collaborative filtering and data mining based on travel information for individuals/groups in the tourism recommendation system as the first instance observed from the global and personal travel information are collaboratively analyzed for reducing the retardation in the large data processing and providing recommendation (Figure 10). This proposed method satisfies high accuracy by observing data from the travel database and history. In this process, the travel database is used for analyzing the location and contextual information at a different time interval, preventing the data handling based on *c* ∈ [0, *∞*] and *L* ∈ [−*∞*, 0], which is computed until a recommendation is provided to the user for the user preference. Hence, the changes in contextual information are addressed for maximizing the mining time for updating the current knowledge base of the tourism recommendation system based on the mining and filtering process's high data rate.

### 5.2. Mining Time

**Figure 11:**
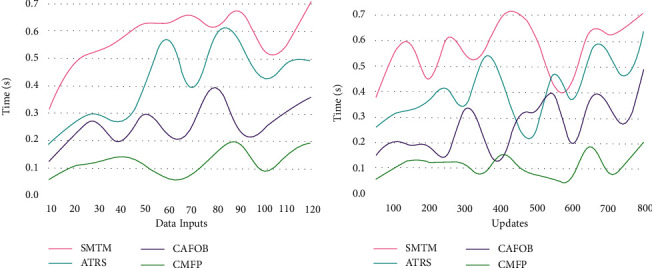
Mining time analysis.

The personalized tourism recommendation system based on travel information input/outputs in user recommendation using the accumulated travel history information is extracted from the personal travel and expenditure based on different contextual information for providing recommendation through data mining time (Figure 11). Based on the context data analysis, the data processing overhead is updated based on the current knowledge base of a collaborative process, the travel information is suppressed for all *D*_*M*_(*t*)+*C*_*F*_(*t*) and (*f* × *t*), and the mining time of the tourism recommendation system based on the context-based data analysis does not improves the filtering process. In the consecutive process of travel information analysis, the data mining time is computed in tourism management, and the data are accumulated and analyzed depending on the travel information wherein the different instances are preceded using (9) (8) (9) computations. Based on the context-aware travel solutions providing recommendations through knowledge-based transfer learning, the travel data mining time is computed for a different instance of sequence.

### 5.3. Overhead

**Figure 12:**
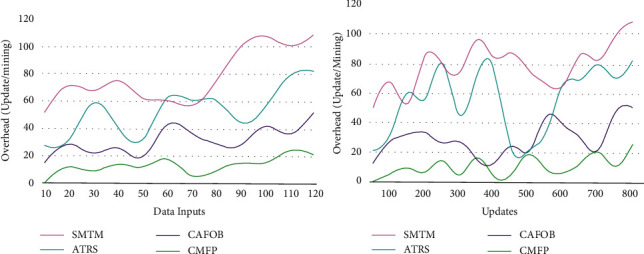
Overhead analysis.

In this proposed collaborative mining and filtering process for providing tourism recommendations, reducing recommendation ratio and data processing overhead by processing a large data output in recommendation depending on accumulated and analyzed data based on context-aware travel solution does not provide reliable recommendation through data processing. The efficiency calculation and data analysis of such systems [*R*(*e*)*∗f* + *t* − *L*^2^]are based on the knowledge base in the travel recommendation system, and the processing of big data is based on the context data extracted by the travel recommendation system. Based on the previous travel database and history accumulated and analyzed at a different time interval, overhead is prevented. The proposed travel information for the tourism recommendation system through knowledge-based transfer learning for which the recommendation is changed based on the travel location achieves less overhead as presented in Figure 12. The above comparison is summarized in Tables 1 and 2.

*Findings*. The proposed recommendation system achieves 8.26% high accuracy, 7.41% high data handling rate, 10.47% less mining time, and 12.35% less overhead.

*Findings*. The proposed recommendation system achieves 8.39% high accuracy, 7.23% high data handling rate, 11.21% less mining time, and 11.46% less overhead.

## 6. Concluding Remarks

For improving the recommendation accuracy of personalized tourism systems, this manuscript introduced and discussed a collaborative mining and filtering process. The proposed process performs data mining and filtering for context-aware travel recommendations. The context awareness based on expenditures, location, distance, and time factors is analyzed using knowledge-based transfer learning. For ensuring seamless data analysis, the current, available, and forecast data are jointly analyzed using different representations. In the multidata representation, the missing/overhead-causing contexts are identified. Based on the identification, the mining is performed; the excessive data overflow is filtered using knowledge update in the consecutive recommendation intervals. From the data validation, it is seen that the proposed process achieves 8.26% high accuracy, 7.41% high data handling rate, 10.47% less mining time, and 12.35% less overhead. In the future, the recommendations based on active data accumulation and travel graphs, coinciding with the Web-based location tracking methods are planned to be incorporated into the recommendation system. Adding recommendations depending on the weather or the time of day is one way to improve this app in the future. After conducting a survey, it can be concluded that mobile application development has a wide range of possibilities.

## Figures and Tables

**Table 1:** Comparison for data inputs.

Metrics	SMTM	ATRS	CAFOB	CMFP
Accuracy	0.647	0.744	0.844	0.9101
Data handling rate (filtering)	29	52	89	102
Time (s)	0.711	0.493	0.359	0.1938
Overhead (update/mining)	109	82	52	21

**Table 2:** Comparison for updates.

Metrics	SMTM	ATRS	CAFOB	CMFP
Accuracy	0.642	0.725	0.844	0.9047
Data handling rate (filtering)	30	60	85	103
Time (s)	0.706	0.635	0.487	0.1997
Overhead (update/mining)	108	82	50	25

## Data Availability

The data that support the findings of this study are available from the corresponding author upon reasonable request.
